# Sustainable Surface Engineering of Lignocellulose and Cellulose by Synergistic Combination of Metal‐Free Catalysis and Polyelectrolyte Complexes

**DOI:** 10.1002/gch2.201900018

**Published:** 2019-06-06

**Authors:** Rana Alimohammadzadeh, Sinke H. Osong, Abdolrahim A. Rafi, Christina Dahlström, Armando Cordova

**Affiliations:** ^1^ Department of Natural Sciences Mid Sweden University Holmgatan 10 851 70 Sundsvall Sweden; ^2^ Department of Chemical Engineering Mid Sweden University Holmgatan 10 851 70 Sundsvall Sweden

**Keywords:** click chemistry, lignocellulose, metal‐free catalysis, selective fluorescent labeling, sustainable polyelectrolyte complex, synergistic surface engineering, water repellent

## Abstract

A sustainable strategy for synergistic surface engineering of lignocellulose and cellulose fibers derived from wood by synergistic combination of metal‐free catalysis and renewable polyelectrolyte (PE) complexes is disclosed. The strategy allows for improvement and introduction of important properties such as strength, water resistance, and fluorescence to the renewable fibers and cellulosic materials. For example, the “green” surface engineering significantly increases the strength properties (up to 100% in *Z*‐strength) of chemi‐thermomechanical pulp (CTMP) and bleached sulphite pulp (BSP)‐derived sheets. Next, performing an organocatalytic silylation with a nontoxic organic acid makes the corresponding lignocellulose and cellulose sheets hydrophobic. A selective color modification of polysaccharides is developed by combining metal‐free catalysis and thiol‐ene click chemistry. Next, fluorescent PE complexes based on cationic starch (CS) and carboxymethylcellulose (CMC) are prepared and used for modification of CTMP or BSP in the presence of a metal‐free catalyst. Laser‐scanning confocal microscopy reveals that the PE‐strength additive is evenly distributed on the CTMP and heterogeneously on the BSP. The fluorescent CS distribution on the CTMP follows the lignin distribution of the lignocellulosic fibers.

Lignocellulose, which is a generic term for describing the main constituents in plants (cellulose, hemicellulose, and lignin), is the most abundant sustainable material on earth and it is the most obvious choice to replace fossil raw materials.^1^ At the moment the forest industry sector has several challenges. Our ways of using paper is changing and the most remarkable changes are the use of graphic paper. The area of packaging is increasing and developing but the challenge is to create fiber‐based materials, which can compete with other types of materials such as plastics. Thoughts that have also increased its importance are about finding new products and materials based on the lignocellulose or cellulose fiber. However, poor moisture resistance and water sensitivity is a major challenge. It is also important to improve the strength of the fibers. This will maximize the efficiency in production of cellulosic materials (e.g., packaging/cardboard production (per square meter)). The stronger fiber‐based material leads to reduced demands of the given cellulosic precursors and allowing for the use of thinner products (e.g., packaging/cardboard). Alternatively, this can be accomplished by replacing thick materials with thinner ones in products and at the end reduce the amount of plant material needed as a starting material. This also leads to decreased energy costs for transportation. Thus, there is a strong need for development of sustainable technology methods, which use environmentally benign and energy‐efficient cellulose and lignocellulose modifications that can address these challenges and reach industrial scale. Herein, we disclose a novel eco‐friendly strategy for improving and installing new properties (e.g., strength and hydrophobicity) of lignocellulose and cellulose fibers by combined use of metal‐free catalysis and sustainable polyelectrolyte complexes.

One strategy of improving properties of pulp derived materials is the use of chemical additives.^2^ In this context, the most common treatment for improving paper strength is the addition of cationic starch (CS) to the fibers.^3^, ^4^, ^5^ However, the absorption of starch can be difficult for some types of chemimechanical pulps (CTMP).^6^ Therefore, alternative treatments have been developed, such as polyelectrolyte multilayer treatment (PEM) concept.^7^ This is an elegant method for modifying a surface, whereby a charged surface is consecutively treated with oppositely charged polyelectrolytes, forming in a layer‐by‐layer technique. A polyelectrolyte is a polymer with its repeating units bearing either an anionic or cationic group. The PEM concept has several applications including modification of surface charge to improving paper strength properties.^8^, ^9^, ^10^, ^11^ One sustainable example of this concept is Wågberg and co‐workers application of layer‐by‐layers of CS and anionic carboxymethylcellulose (CMC) for enhancing the strength properties of papers formed from different pulps and CTMP.^11^

Cordova and Hafren have shown that the use of catalysis^12^ in combination with polysaccharides containing carboxyl groups (e.g., CMC and pectin) can improve the strength properties of cellulose or lignocellulose‐based paper sheets by promoting covalent cross‐linking and esterification (**Figure** 1).^13^ This biomimetic approach for improvement of fiber‐properties can also be performed at a multiton scale.^14^

**Figure 1:**
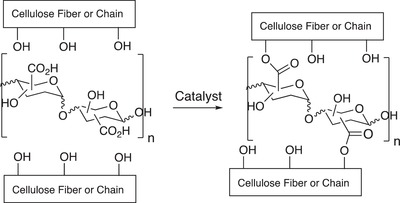
Catalytic direct cross‐linking and esterification of cellulose and lignocellulose pulps.^13^

In this report, a sustainable strategy for significantly improving the strength properties and water repellence of sheets made from lignocellulose or cellulose pulps using a synergistic combination of metal‐free catalysis and sustainable polyelectrolyte complexes is presented. A simple and scalable mixing protocol was applied for paper sheet manufacturing. Moreover, a selective polysaccharide color marking method is disclosed and used for modifying and showing the distribution of the CS on the lignocellulosic and cellulosic fibers.

The mixing of polyanions and polycations in aqueous solution can form polysalts in a complexation process driven by a process closely linked to self‐assembly. The general conception is that the main driving force for the polyelectrolyte complex formation is the gain in entropy caused by the release of low molecular weight counterions associated to the polyelectrolytes.^15^ We began our experiments by preparing different sustainable polyelectrolyte complexes by mixing CS or chitosan with CMC and then mixing them with or without an organic catalyst. Next, this PE‐complex mixture (2 wt%) was added to either CTMP or BSP and sheets were constructed using a Rapid‐Koethen sheet former (**Tables** 1 and **2**). We found that the properties of the CTMP sheets were significantly improved by the addition of a PE complex made up of CS and CMC in the presence of an organic α‐hydroxy acid catalyst (Table 1). In this context, the best results were obtained with citric acid (CA) and tartaric acid (TA). Thus, we chose to use CA as the catalyst for further investigation. In Table 1, it is possible to see that adding a modest catalyst loading (0.4–0.6 wt%) to the CS,CMC PE‐complex mixture increased the tensile index, the tensile strength in N m^−1^ divided by grammage, with up to 57% and the *Z*‐strength, the strength of paper and board as related to the force and measured by means of tension applied to the surfaces of the test sample, with up to 100% (Entries 4 and 5, **Figure** 2). The density was not significantly increased. In the cases when a Chitosan, CMC PE complex was used for CTMP, the strength properties were significantly improved. However, a lower catalyst effect was observed (entries 1,6–9).

**Table 1:** The tensile index and *Z*‐strength of sheets made from CTMP

Entry	PE complex [2 wt%]	Cat. [0.6 wt%]	δ [kg m^−3^]	TI [kN m kg^−1^]	*Z*‐strength [kN m^−2^]
1	–	–	446	27.9	219
2	CS, CMC	–	458	37.8	327
3	CS,CMC	tartaric acid	455	44.2	437
4	CS, CMC	acetic acid	447	36.7	353
5	CS, CMC	citric acid	460	41	438
6	Chit, CMC	–	466	35.2	290
7	Chit, CMC	citric acid	469	34.7	288
8	Chit, CMC[^a)^]	citric acid	458	35.8	304
9	Chit, CMC[^a)^, ^b)^]	citric acid	461	36.5	311

^a)^ Heating the sheets for 30 min at 110 °C after sheet forming

^b)^ 0.4 wt% catalyst was used.

**Table 2:** The tensile index and *Z*‐strength of sheets made from BSP

	PE complex [2 wt%]	Cat. [0.6 wt%]	δ [kg m^−3^]	TI [kN m kg^−1^]	*Z*‐strength [kN m^−2^]
1	–	–	400	8.4	234
2	CS, CMC	–	416	11.4	260
3	CS,CMC	citric acid	431	12.9	292
4	CS, CMC[^a)^]	citric acid	421	12.6	306
5	Chit, CMC	–	417	10.6	261
6	Chit, CMC[^a)^]	–	415	11	288
7	Chit, CMC	citric acid	426	9.9	248
8	Chit, CMC[^a)^]	citric acid	419	11	223

^a)^ Heating the sheets for 30 min at 110 °C after sheet forming.

**Figure 2:**
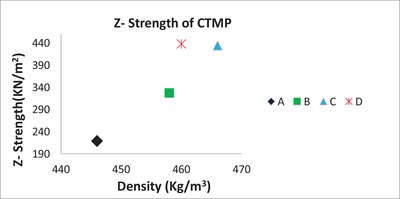
*Z*‐strength of CTMP as a function of density. A) CTMP sheet. B) CTMP‐CS‐CMC sheet, C) CTMP‐CS‐CMC‐CA (0.4 wt%) sheet, and D) CTMP‐CS‐CMC‐CA (0.6 wt%). CA = citric acid.

Next, we performed the same catalytic polyelectrolyte treatments on BSP (Table 2). The results demonstrated that the catalytic treatment was successful and the tensile index was increased with up to 54% and *Z*‐strength with up to 31% respectively (entries 2–4, **Figure** 3). We also found a significant increase in strength properties when using chitosan instead of CS as the cationic polyelectrolyte. However, the use of catalytic amounts of citric acid did not further improve the properties.

**Figure 3:**
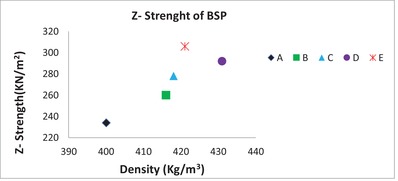
*Z*‐strength of BSP as a function of density. A) BSP sheet, B) BSP‐CS‐CMC, C) BSP‐CS‐CMC‐H, D) BSP‐CS‐CMC‐ CA (0.6 wt%), E) BSP‐CS‐CMC‐ CA (0.6wt%)‐H, H = Heating the sheets for 30 min at 110 °C after sheet forming. CA = citric acid.

When comparing the strength properties of CTMP with BSP, the strength improvement was higher for the lignocellulosic CTMP. In order to elucidate how the PE complex is distributed on the sheets, we decided to covalently mark the CS component with a fluorescent molecule.^16^, ^17^, ^18^ In order to that, an combined catalysis and click‐chemistry strategy was envisioned using organocatalytic direct esterification followed by a suitable click reaction (**Scheme** 1).^19^ Thus, we performed a tartaric acid‐catalyzed direct esterification of thioglycolic acid (TGA) on CS.^20^ The reaction was successful as demonstrated by IR and NMR analysis. Next, a thiol‐ene click reaction was performed between the TGA esterified CS (CS‐TGA) and a synthesized 5‐ and 6‐(carboxamidoallyl)tetramethylrhodamine (allyl‐TAMRA) (Scheme 1). The reaction gave the corresponding pink CS‐TGA‐TAMRA in high yield. The reaction does also work with 5‐ and 6‐(carboxamidoallyl)fluorescein (allyl‐FAM) and cinchona alkaloids such as quinidine. However, FAM and quinidine has a similar fluorescence spectrum as to lignin and was therefore not chosen for further studies on the CTMP. Instead the CS‐TGA‐quinidine was used for the studies on BSP.

**Scheme 1:**
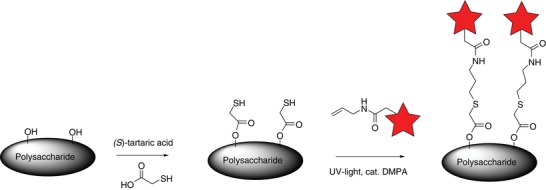
Catalytic fluorescent labelling of polysaccharides with allyl amide dyes and fluorescent molecules.


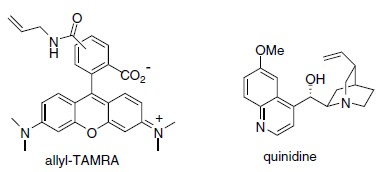


With these results in hand, we could make a CS‐TGA‐TAMRA,CMC‐PE‐complex and then mix them together with a catalytic amount of CA and CTMP or BSP to form the corresponding pulp‐mixtures for sheet fabrication by the Rapid Koethen machine. Different sheets were prepared and analyzed by a confocal laser scanning microscopy using specific emission ranges for TAMRA (568–685 nm) and lignin (410–505 nm) (**Figure** 4).

**Figure 4:**
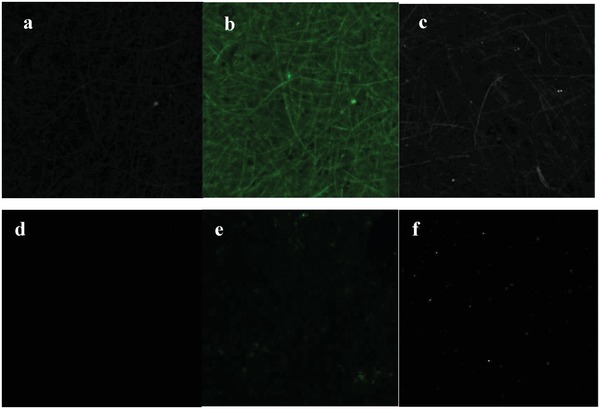
a) CTMP sheet (λ = 561 nm). b) CTMP sheet (λ = 405), c) CA‐treated CS‐TGA‐TAMRA,CMC‐CTMP (λ = 561 nm), d) BSP sheet (λ = 561 nm), e) BSP sheet (λ = 405 nm), f) CA‐treated CS‐TGA‐TAMRA,CMC‐BSP sheet (λ = 561 nm).

The confocal laser scanning microscopy clearly revealed that the CS component of the PE complex was evenly distributed in the CS‐TGA‐TAMRA,CMC‐CTMP (Figure 4a) and heterogeneously in the CS‐TGA‐TAMRA,CMC‐BSP (Figure 4f). The CS‐TGA‐TAMRA,CMC‐CTMP‐sheets light emission follows the fiber patterns of the CTMP. This pattern is in line with the pattern of lignin as can be visualized by its auto‐florescence (Figure 4b). The CS‐component on the CS‐TGA‐TAMRA,CMC‐BSP‐sheets was observed as dots (Figure 4f) at a wavelength of 561 nm. This was also the case for the CS‐TGA‐quinidine, CMC‐BSP sheets (**Figure** 5, λ = 405 nm).

**Figure 5:**
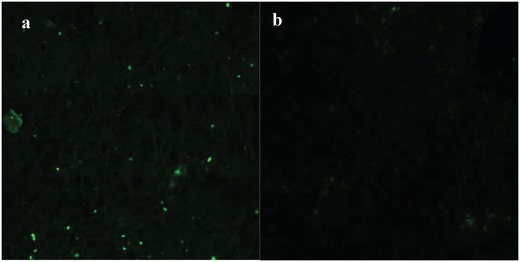
a): CA‐treated CS‐TGA‐quinidine,CMC‐BSP sheet (λ = 405 nm), b) BSP sheet (λ = 405 nm).

The distribution pattern was not dependent on the catalyst since they were the same for sheets just treated with CS‐TGA‐TAMRA,CMC PE‐complex without addition of CA (see the Supporting Information). Thus, the significant difference in distribution as well as binding of the CS‐component is likely due to the presence of lignin, which is evenly distributed in the CTMP fibers. It could also explain that the addition of the sustainable PE complex had a larger effect on the strength properties of the CTMP‐sheets as compared to BSP‐sheets. We also investigated our CTMP and BSP sheets with scanning electron microscopy (SEM). The SEM analysis revealed that a slight densification had occurred when adding the polyelectrolyte complex (**Figure** 6). This is in agreements with the measured experimental values (Figure 2).

**Figure 6:**
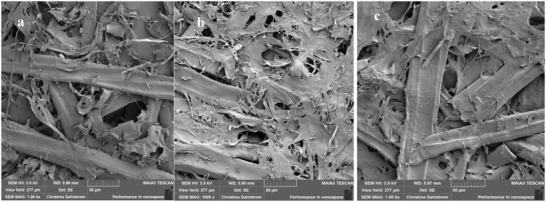
SEM images at 1000 times magnification. a) CA (0.6 wt%)‐treated CS,CMC‐CTMP. b) CTMP sheet. c) CS,CMC‐CTMP.

Having established the possibility of increasing the strength properties of the lignocellulose and cellulose sheets, we began to address the improvement of their hydrophobicity. All of the above sheets, had no water resistance and a contact angle of 0°. Thus, organocatalytic silylations using hexadecyltrimethoxysilane as the hydrophobizing agent and tartaric acid (5 mol%) as the nontoxic catalyst were performed on the CTMP‐ and BSP‐sheets. To our delight, the sheets got very hydrophobic with contact angles ranging in the 127° for the CTMP‐sheets and 137° for the BSP‐sheet, respectively (**Figure** 7).

**Figure 7:**
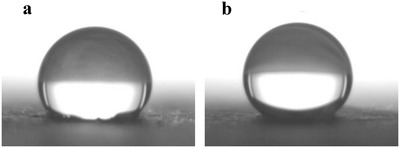
a) Water droplet on CTMP. b) Water droplet on BSP.

In summary, we have developed a sustainable strategy for surface engineering properties (e.g., strength, water resistance and fluorescence) of lignocellulose and cellulose fibers by using a synergistic combination of metal‐free catalysis and PE complexes. This was exemplified by fabricating sheets made from chemimechanical pulp and bleached sulphite pulp using sustainable PE complexes derived from CS or chitosan, CMC and a metal‐free nontoxic catalyst. The catalytic surface engineering significantly increased the strength properties of the assembled sheets (up to 100%, *Z*‐strength for CTMP). We also developed a catalytic selective color marking method for polysaccharides based on metal‐free catalysis and thiol‐ene click chemistry. Thus, a fluorescent molecule was installed on the CS component of the PE complex. Next, fluorescent PE complexes were prepared and used for surface engineering of the cellulosic fibers. The usefulness of the method was shown by investigating the distribution of the PE complex on the CTMP and BSP derived sheets. Confocal laser scanning microscopy revealed an even distribution of the PE complex on the CTMP fibers, which follows the fiber‐pattern. This was not the case for the BSP where an uneven distribution was detected. The even distribution of the PE complex on the CTMP fibers is attributed to the presence of nonpolysaccharide components of the lignocellulosic fibers. Thus, the presence of lignin contributes to an even spread of PE complex on the CTMP sheets. We also linked the sustainable fiber surface engineering with an organocatalytic silylation reaction, which provided the corresponding water‐repellent CTMP‐sheets and BSP‐Sheets. The nontoxic and sustainable chemistry presented here, which allows for significant strength improvement of lignocellulose and cellulose fibers, is important since it can lead to reduction of a given renewable plant starting material (e.g., spruce, pine), which is used in large amounts for the synthesis of biodegradable products (e.g., packaging, cardboard). It can also improve the properties of recycled cellulose‐pulp from these products.^21^ The improvement of water resistance enables the cellulosic materials to compete in areas where today fossil‐based plastics are used. Taken as a whole, the surface engineering is well in line toward developing a circular bio‐economy. Further fluorescence microscopy studies as well investigation of other strength additives, lignocelluloses and nanocelluloses are ongoing in our laboratories. Large‐scale factory trials at a ton‐scale are planned and will also be performed.

## Conflict of Interest

The authors declare no conflict of interest.

## Supporting information

Supplementary
