# Supplementary material for: Sustainable Surface Engineering of Lignocellulose and Cellulose by Synergistic Combination of Metal‐Free Catalysis and Polyelectrolyte Complexes
Source: Glob Chall. 2019 Jun 6;3(7):1900018. doi: 10.1002/gch2.201900018 (PMC6607423; doi:10.1002/gch2.201900018)
Supplement: Supplementary file 1 — Supplementary [file GCH2-3-1900018-s001.pdf]

Copyright WILEY-VCH Verlag GmbH & Co. KGaA, 69469 Weinheim, Germany, 2019.

Global Challenges
Open Access

Supporting Information

for *Global Challenges,* DOI: 10.1002/gch2.201900018

Sustainable Surface Engineering of Lignocellulose and Cellulose by Synergistic Combination of Metal-Free Catalysis and Polyelectrolyte Complexes

*Rana Alimohammadzadeh, Sinke H. Osong, Abdolrahim A. Rafi, Christina Dahlström, and Armando Cordova**

Sustainable Surface Engineering of Lignocellulose and Cellulose by Synergistic Combining Metal-free Catalysis and Polyelectrolyte Complexes

Rana Alimohammadzadeh [a], Sinke Henshaw [b], Abdolrahim A. Rafi [a], Christina Dahlström [b], Armando Cordova [a]*

[a]Department of Natural Sciences, Mid Sweden University, Holmgatan 10, 851 70 Sundsvall, Sweden [b]Department of Chemical Engineering , Mid Sweden University, Holmgatan 10, 851 70 Sundsvall, Sweden
E-mail: armando.cordova@miun.se

Table of contents

General 3

Procedure for making handmade sheets of bleached sulphite pulp (BSP) 3

.... 4

Fig. S1: Rapid-Köthen sheet former 4

Procedure for handmade sheets of lignocellulosic CTMP. 4

Typical procedure for chemically modification of BSP or CTMP using a combination of PE-complex and organic acid catalyst: 4

Typical procedure for fluorescent marking of lignocellulosic hand-sheets of BSP or CTMP: 5

Procedure for functionalizing cationic starch with thioglycolic acid (CS-TGA): 5

Procedure for synthesis of Allyl-TAMRA: 5

Procedure for thiol-ene click reaction with modified cationic starch and Allyl-TAMRA (CS-TGA-TAMRA): 6

Fig. S2. CS-TAMRA 6

Procedure for thiol-ene click reaction between modified cationic starch and quinidine (CS-TGA-quinidine): 7

Fig. S3: SEM images with 2500 magnification of BSP, a: BSP-sheet b: CS,CMC-BSP c: Citric acid treated (0.6 wt%) CS, CMC-BSP-sheet. 8

Fig. S4. SEM images with 1000 magnification of (a) CTMP-sheet. (b) CS,CMC-CTMP-sheet. (c) Citric acid treated (0.6 wt%) CS,CMC-CTMP-sheet 9

Fig. S5: ^{1}H-NMR spectrum of Allyl-TAMRA.SE 10

Fig. S6: ^{13}C-NMR spectrum of Allyl-TAMRA.SE 11

Fig. S7-Fig. S20. 12-25

General

Chemicals and solvents were purchased from commercial suppliers. Commercial reagents were used as purchased without any further purification. Infrared spectrum was recorded with a Varian 610 IR microscope coupled to a Varian 670-IR spectrometer. Surface images were obtained using a field emission scanning electron microscope (MAIA3 model 2016, TESCAN). Secondary electron images were generated using 3 kV accelerating voltage. Prior to imaging the samples, were coated with a 5 nm layer of Iridium. Laser scanning microscopy was performed with a Carl Zeiss LSM 880 with objective: EC plan- Neofluar 10x/0.3. Filter for TAMRA: 568-685 nm, Laser for TAMRA: 561 nm; Filter for Lignin: 410-505 nm, Laser for TAMRA: 405 nm; Filter for quinidine: 410-505 nm, Laser for quinidine: 405 nm.

The pulps, which were used in this work, were commercial available sulphite softwood dissolving pulp (Domsjö Fabriker AB, Örnsköldsvik, Sweden) with a very low content of hemicellulose (<5%) and lignin (<1%) with 15% consistency (mass of dry pulp/ mass of dry pulp + mass of rest of the suspension) and CTMP with 47% consistency was obtained from Billerud-Korsnäs Rockhammar mill and made from a composition of Scots pine (pinus sylvestris) and Norway spruce (picea abies). The trade name of the CS used here (DS, 0.065) was Solbond PC 65 (SOLAM GmbH, Emlichheim, Germany). The trade name for the CMC used here was Finnfix 5000 CMC (CPKelko a HUBER COMPANY). The tartaric acid was stored in a desiccator over phosphorus pentoxide. Disintegration was done with (Lorentzen & Wettre, type 961699) according to the ISO 5263-1. International standard (ISO 5269-2) was followed for preparation of laboratory sheets with Rapid-Köthen sheet former (RK) (Blattbildner-SheetFormer type PK3-KWT).

Procedure for making handmade sheets of bleached sulphite pulp (BSP)

The various laboratory hand-sheets were made according to the the ISO 5269-2 method using a Rapid-Köthen sheet former (Figure S1). The suspension of sulphite pulp (50 gram dry mass) dissolved in water (2 L) was stirred for 15 minutes and was next disintegrated with disintegration machine. After that, the mixture was diluted with water to 7.5 - 8 kilogram total weight and stirred for 10 minutes. The resulting mixture was transferred into the Rapid Köthen sheet former

and hand-sheets were made. The sheets were dried at 95˚C at an applied pressure of 96 kPa for 10 min; paper testing was performed in the standard testing climate described in ISO 187, i.e., 23 ˚C and 50% relative humidity.

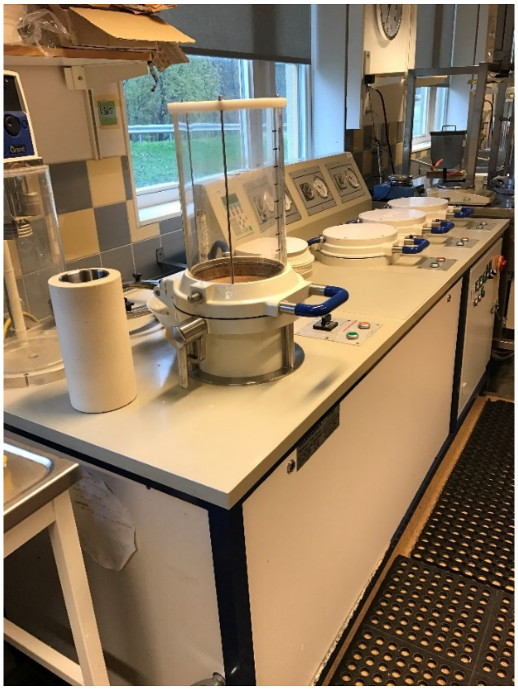

Fig. S1: Rapid-Köthen sheet former

Procedure for handmade sheets of lignocellulosic CTMP.

A suspension of CTMP (50 gram of dry mass) was dissolved in hot water (2L) and mixed for 15 minutes. Next, hot disintegration was performed at 85 °C, which was followed by dilution of the mixture with water to obtain a total weight of 8 - 8.5 Kg. After stirring for 10 minutes, the mixture was transferred into Rapid Kothen sheet former and hand-sheets were made. The sheets were dried at 95˚C at an applied pressure of 96 kPa for 10 min; paper testing was performed in the standard testing climate described in ISO 187, i.e., 23 ˚C and 50% relative humidity.

Typical procedure for chemically modification of BSP or CTMP using a combination of PE-complex and organic acid catalyst:

A suspension of BSP or CTMP (50 gram calculated of dry mass) was mixed in water (2 L) for 15 minutes and next disintegrated. The resulting mixture was diluted by addition of water to

reach 7.5-8 Kg in total weight followed by mixing for 5 minutes. Next, the solutions of cationic starch (50 g, 10g/1L cooked in water, 1%wt to pulp), CMC solution (0.5 g dissolved in 500 ml water, 1%wt to pulp) and citric acid (300 mg, 0.6%wt to pulp), which had been homogenized, was added to the pulp solution and the resulting mixture was mixed for 10 minutes. the mixture was transferred into Rapid Köthen sheet former and hand-sheets were made. The sheets were dried at 95°C at an applied pressure of 96 kPa for 10 min; paper testing was performed in the standard testing climate described in ISO 187, i.e., 23 °C and 50% relative humidity

Typical procedure for fluorescent marking of lignocellulosic hand-sheets of BSP or CTMP:
Sulphite pulp (25g) or CTMP (25 g based on calculated dry mass) was mixed in water (2L). After mixing for 15 minutes, the pulp was disintegrated and the resulting mixture was diluted with water to reach a total weight of 3.5–4 Kg and then stirred for 5 minutes. Next, a solution of TAMRA-modified cationic starch (CS-TGA-TAMRA) (0.25 g cooked in 50 mL of water, 1%wt to pulp), CMC (0.25 g dissolved in 250 mL of water, 1%wt to pulp) and citric acid (150 mg, 0.6%wt to pulp) was homogenized and then added to the pulp solution and mixed for an additional 10 minutes. The mixture was thereafter transferred to the Rapid Köthen sheet former and hand-sheets were made. The sheets were next dried at 95°C at an applied pressure of 96 kPa for 10 min

Procedure for functionalizing cationic starch with thioglycolic acid (CS-TGA):
To an oven-dried round-bottomed flask charged with cationic starch (2.5 g, 1.0 equiv.) in dried toluene (40 mL), tartaric acid (112 mg, 0.75 mmol, 5 mol %) and thioglycolic acid (69mg, 0.5equiv.) was added in written order. Next, the reaction mixture was refluxed under nitrogen. After 24 h, the reaction flask was cooled down to room temperature and the modified cationic starch, was washed by acetone using Soxhlet extraction for 18h. The modified Cationic Starch was dried under vacuum. IR: ν 3319, 2926, 2285, 1720, 1296, 1144, 1074, 992, 761, 572, 520cm^{-1}.

Procedure for synthesis of Allyl-TAMRA:
To the solution of allyl amine (1.14mg, 0.02mmol, 2eq.) in 1ml acetonitrile, TAMRA, SE (5mg, 0.01mmol, 1equiv.) was added and stirred in room temperature in darkness for 24 hours. Then it was purified by flash chromatography using CH_2Cl_2: Methanol (6:1) and isolated yield was 90%.

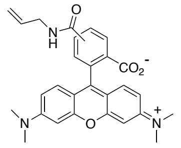

^{1}H NMR (MeOD-d4, 500 MHz) δ: 8.2 (t, *J*=8.5,1H), 8.16-8.12(m, 1H), 7.29(s,broud, 1H), 7.25(t, *J*=9.4, 2H), 7.06- 7.03(m, 2H), 6.97-6.95(m,2H), 5.98-5.89(m, 1H), 5.24(dd, *J*=7, *J*=1Hz, 1H), 5.14(dd, *J*=7, *J*=1Hz, 1H), , 4.01(d,broud, 2H), 3.31(s, 12H) 2.1(s,1H). **^{13}C NMR (MeOD-d4, 125 MHz)** δ: 208.6, 169.7, 169.8, 160.4, 157.7, 157.4, 135.6, 133.9, 133.0, 131,1, 130.1, 128.4, 128.1, 115.0, 113.7, 113.5, 95.9, 56.0, 42.0, 39.4, 29.2. **HRMS (ESI^{+}) [M+H]$^{+}$** calcd: 470.2074, found: 470.2081.

Procedure for thiol-ene click reaction with modified cationic starch and Allyl-TAMRA (CS-TGA-TAMRA):

To a scintillation vial (20 mL) containing 600 mg of modified Cationic Starch with Thioglycolic acid, allyltamra (5 mg, 0.01 mmol) and 3 ml DMF, DMPA (6 mg, 0.006 mmol, 10%w) was added. The reaction was irradiated for 24 hours using an UV-lamp (UV-Bulb, TL20W/12 and 20 W). The mixture was washed with Soxhlet extraction by acetone. After drying under vacuum overnight, the marked Cationic Starch with TAMRA was achieved (figure2). IR: ν 3306, 2925, 1721, 1658, 1631, 1293, 1146, 1076, 858, 762, 571,520 cm^{-1}.

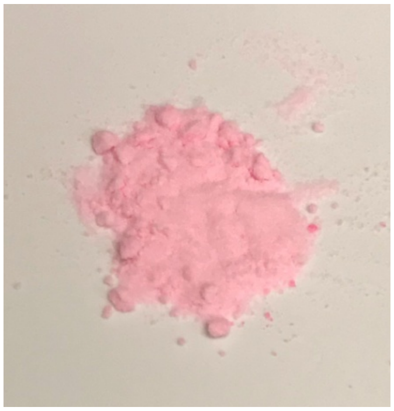

Fig. S2. CS-TAMRA

Procedure for thiol-ene click reaction between modified cationic starch and quinidine (CS-TGA-quinidine):

To a scintillation vial (20 mL) containing 2 g of thioglycolic acid-modified cationic starch, quinidine (100 mg, 0.3 mmol), DMF (2 mL), DMPA (2 mg, 0.006 mmol, 2%mol) were added. Next, the reaction mixture was irradiated using an UV-lamp (UV-Bulb, TL20W/12 and 20 W). After 24h, the solid starch material was washed with Soxhlet extraction using acetone for 24 h. Next, drying under vacuum gave the solid UV-active cationic starch derivative. . IR: ν 3310, 2924, 2287, 1721, 1294, 1146, 1075, 857, 761, 701, 572, 521, 401cm^{-1}.

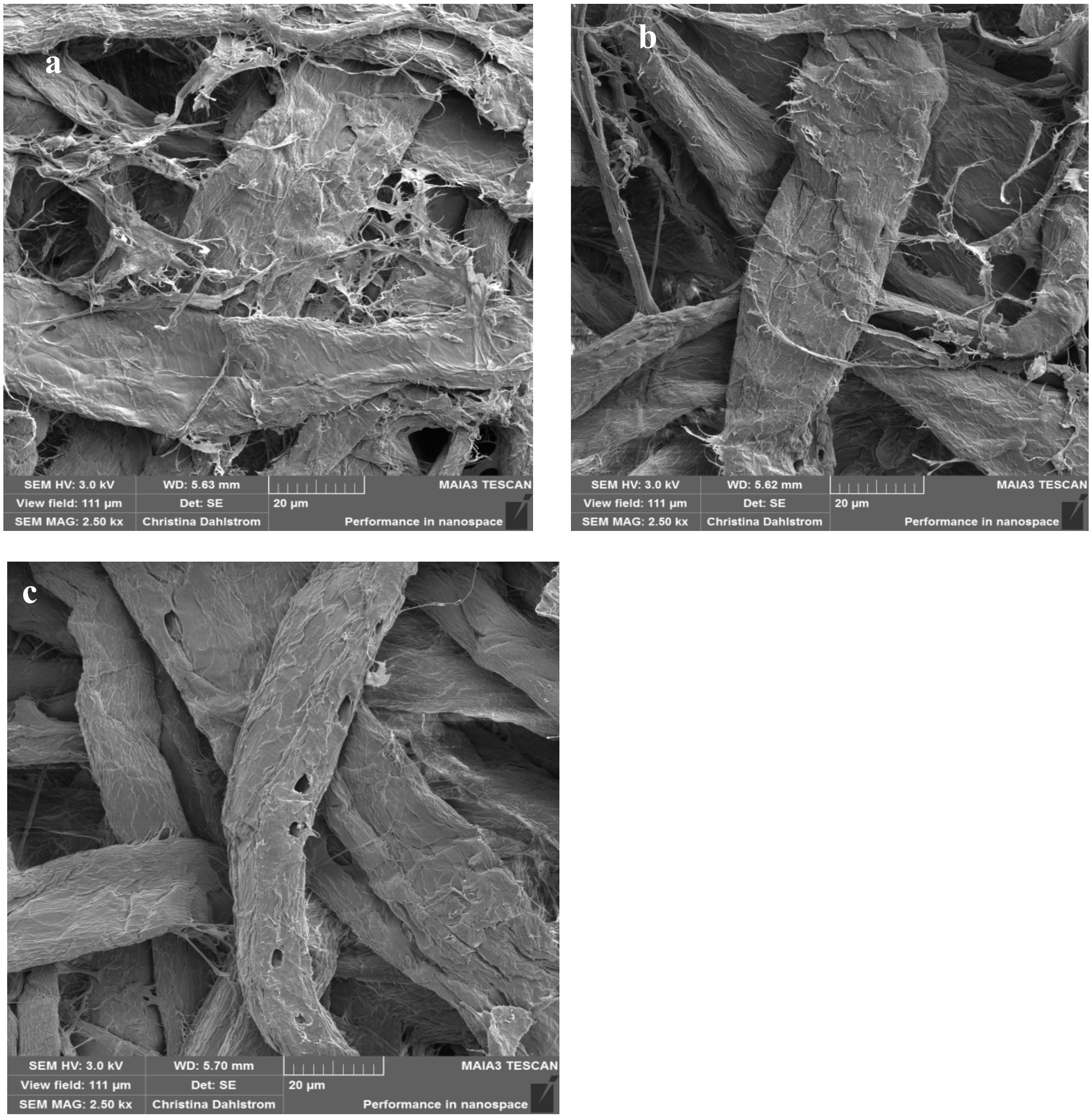

Fig. S3: SEM images with 2500 magnification of BSP, a: BSP-sheet b: CS,CMC-BSP c: Citric acid treated (0.6 wt%) CS, CMC-BSP-sheet.

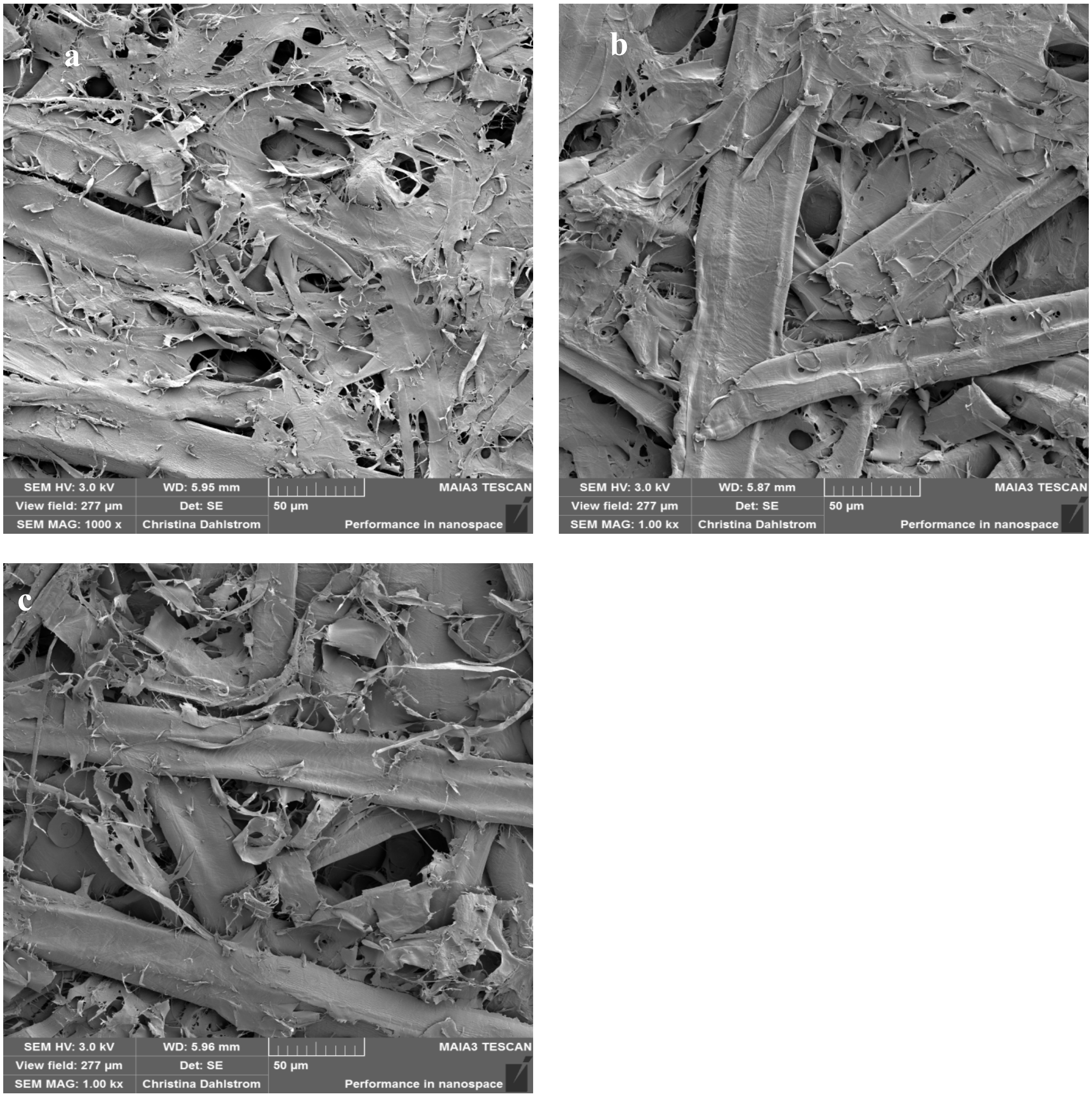

Fig. S4. SEM images with 1000 magnification of (a) CTMP-sheet. (b) CS,CMC-CTMP-sheet. (c) Citric acid treated (0.6 wt%) CS,CMC-CTMP-sheet.

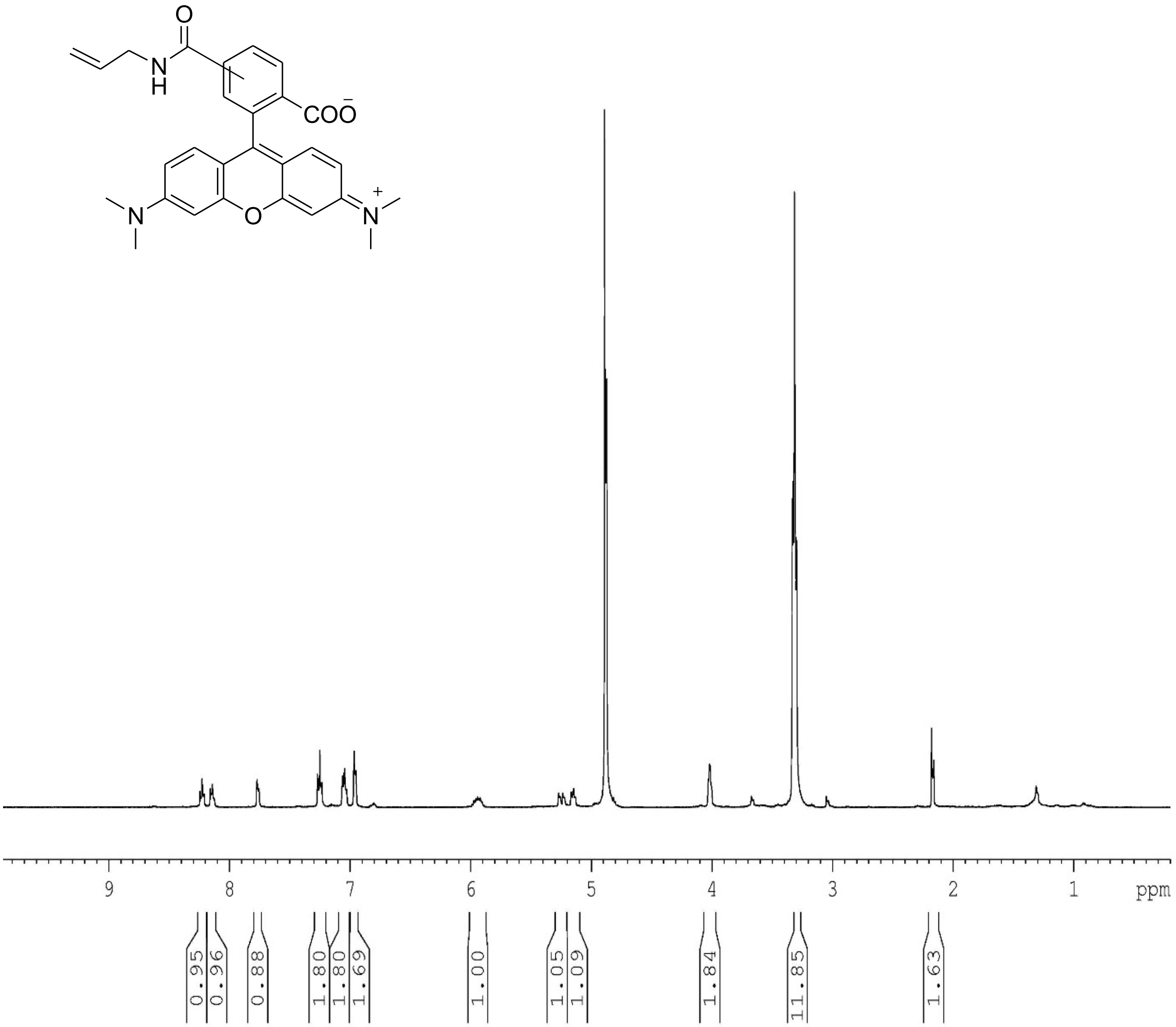

Fig. S5: ^{1}H-NMR spectrum of Allyl-TAMRA.SE

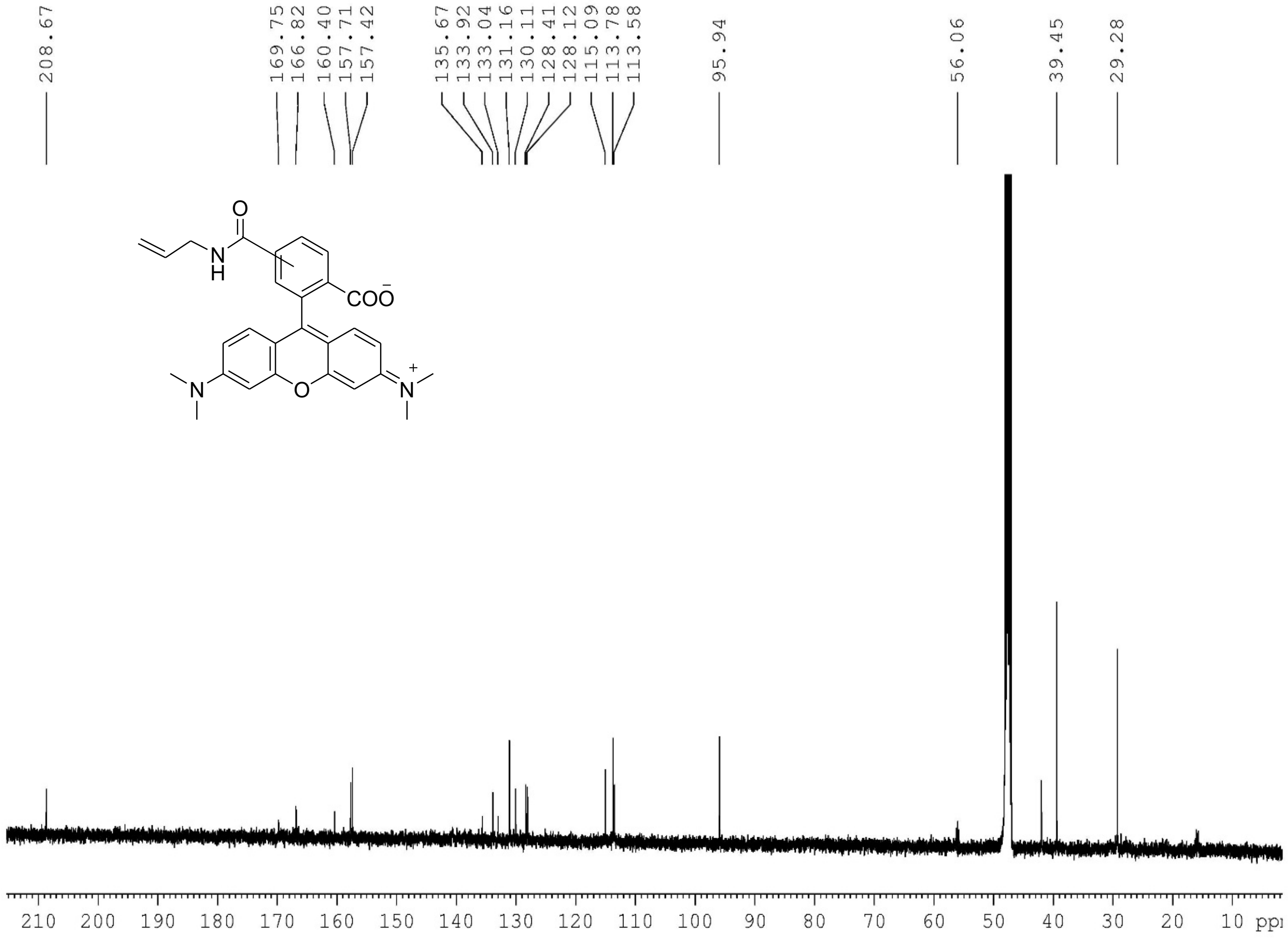

Fig. S6: ^{13}C-NMR spectrum of Allyl-TAMRA.SE

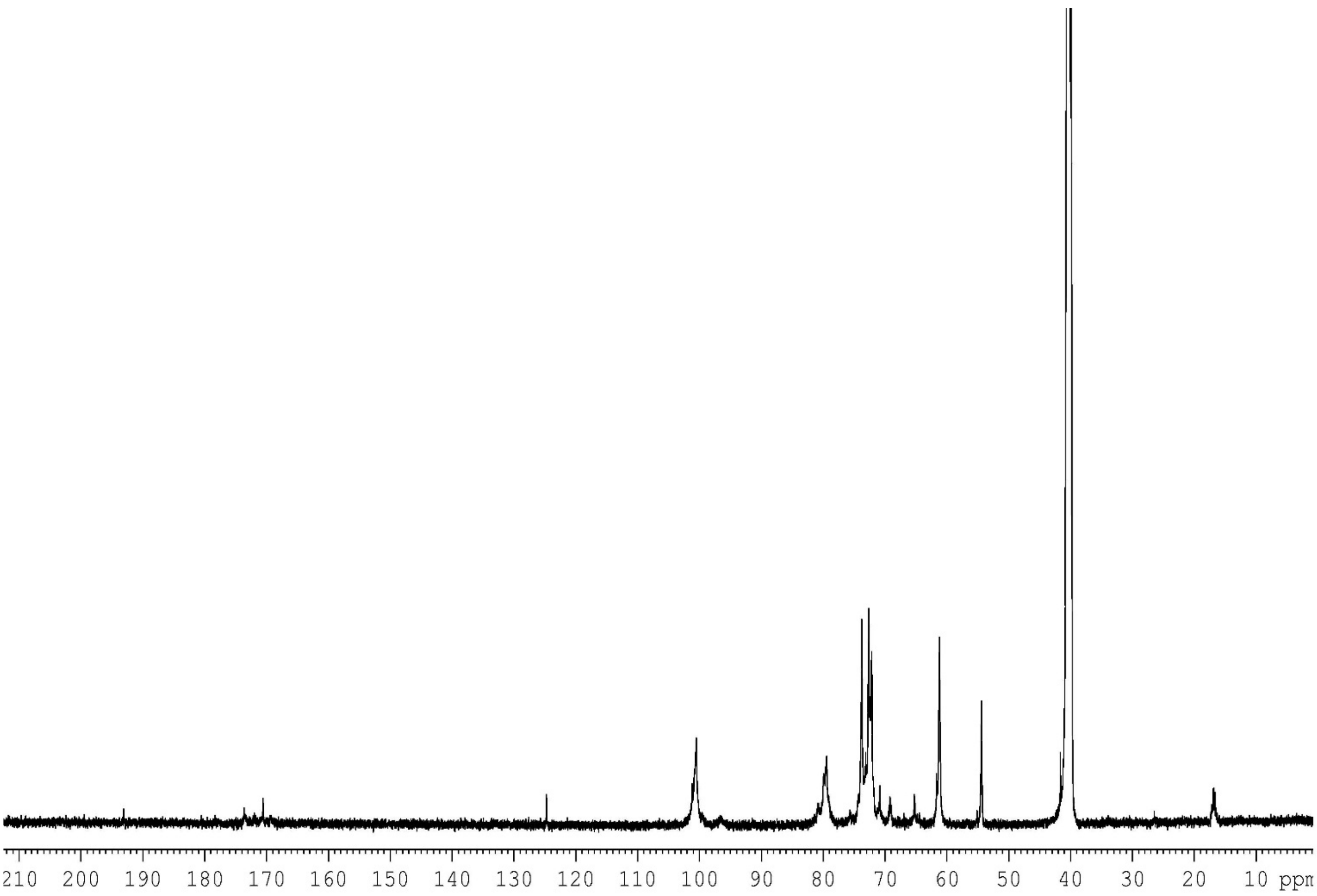

Fig. S7: ^{13}C-NMR spectrum of modified Cationic Starch with Thioglyconic acid

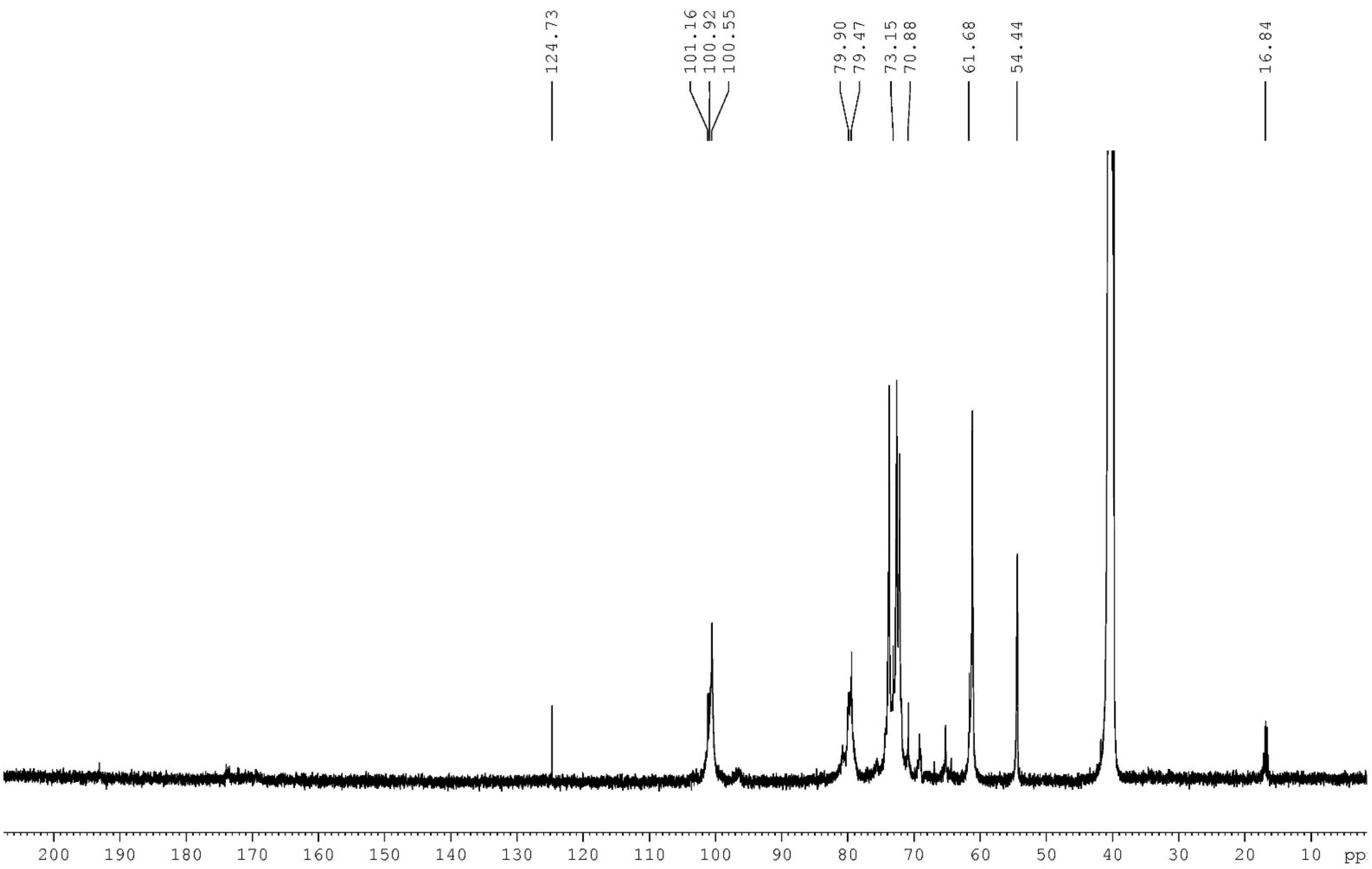

Fig. S8: 13C-NMR spectrum of modified Cationic starch with Allyl-TAMRA.

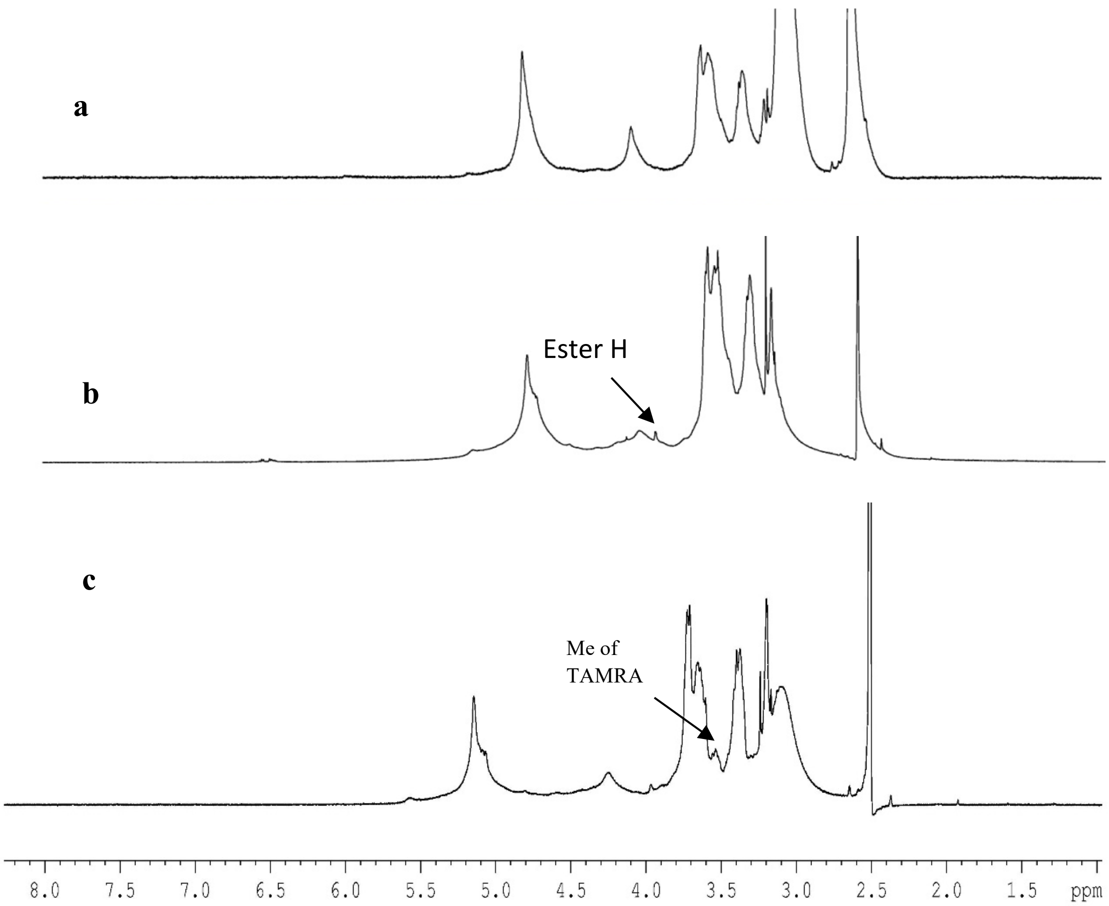

Fig. S9. ^{1}H NMR (DMSO-d6, 75°C) spectra of (a) cationic starch (CS). (b) CS esterified with thioglycolic acid (CS-TGA), (C) Cationic Starch-TGA-TAMRA.

a

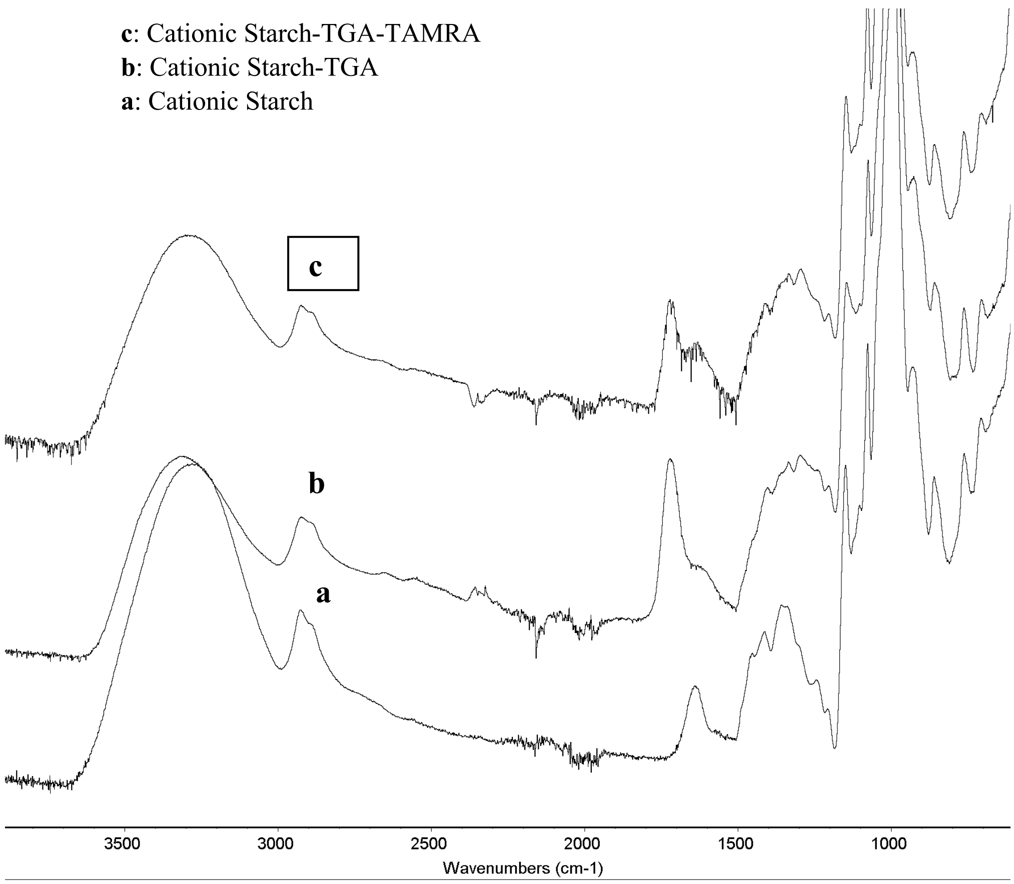

Fig. S10. IR spectra of (a) Cationic starch (CS). (b) CS-TGA. (c) CS-TGA-TAMRA.

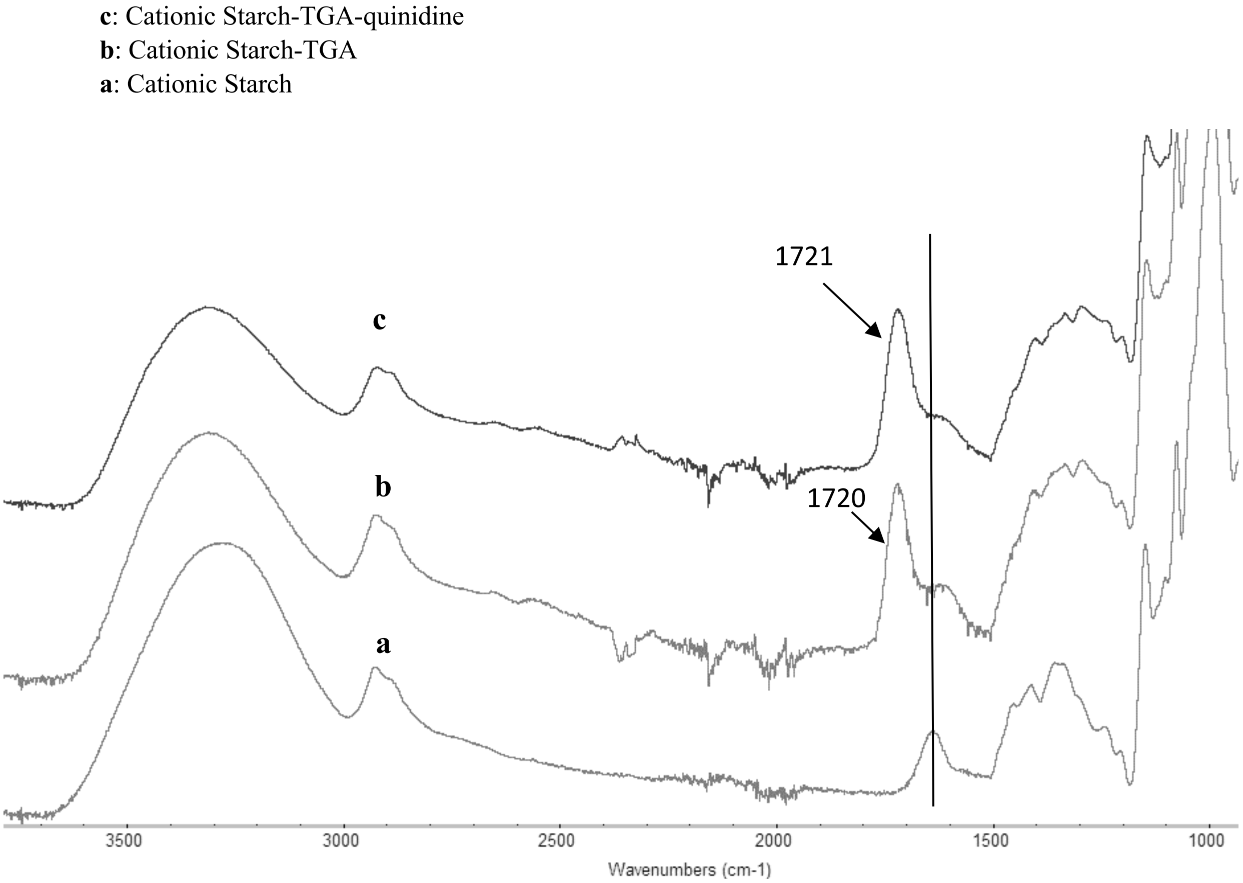

Fig. S11. IR spectra of (a) Cationic starch (CS). (b) CS-TGA. (c) CS-TGA-quinidine.

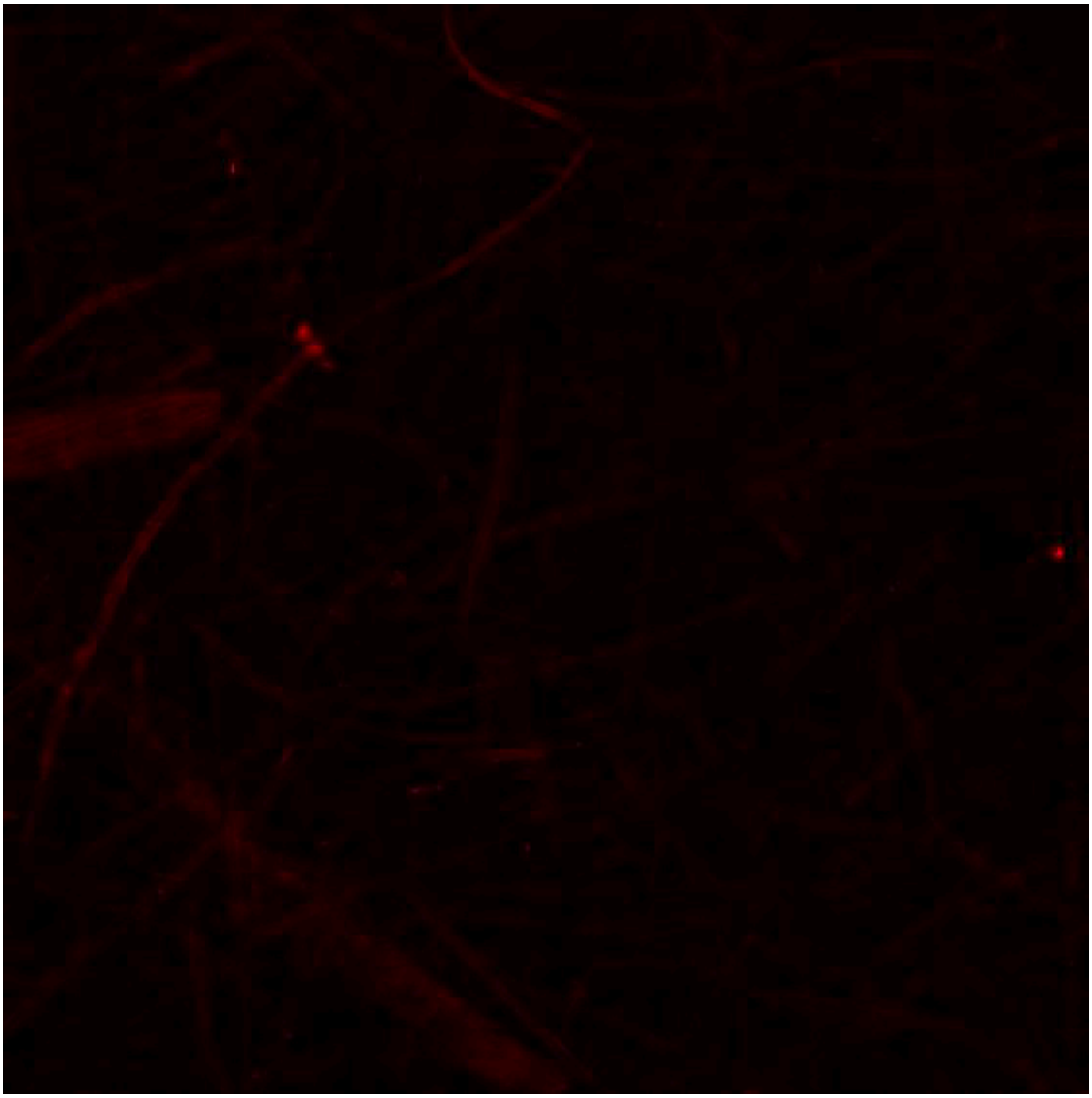

Fig. S12. Confocal laser scanning microscopy of CS-TGA-TAMRA,CMC-CTMP sheet. (λ = 561 nm

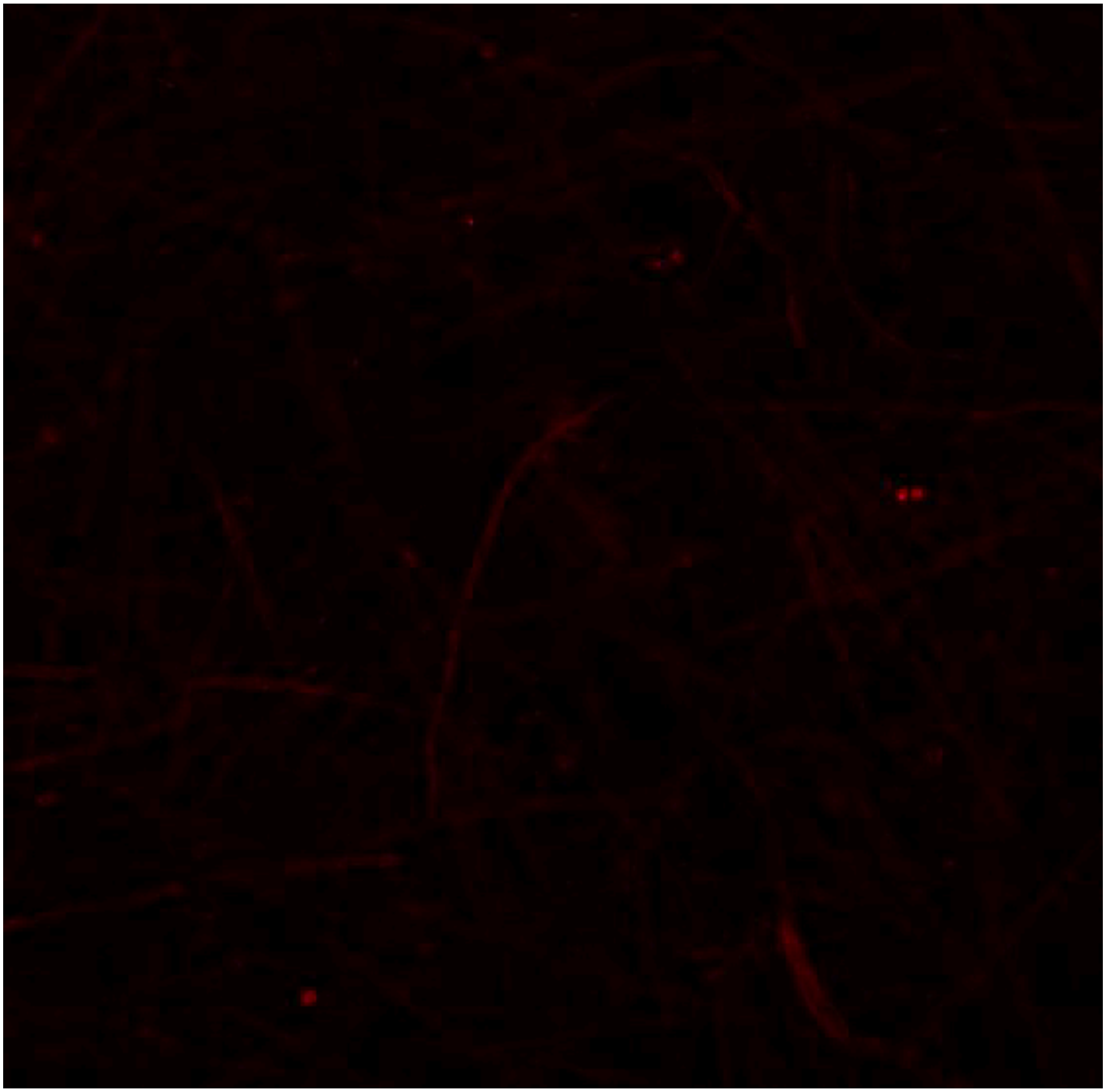

Fig. S13. Confocal laser scanning microscopy of CA-treated (0.6 wt%) CS-TGA-TAMRA,CMC-CTMP sheet. (λ = 561 nm).

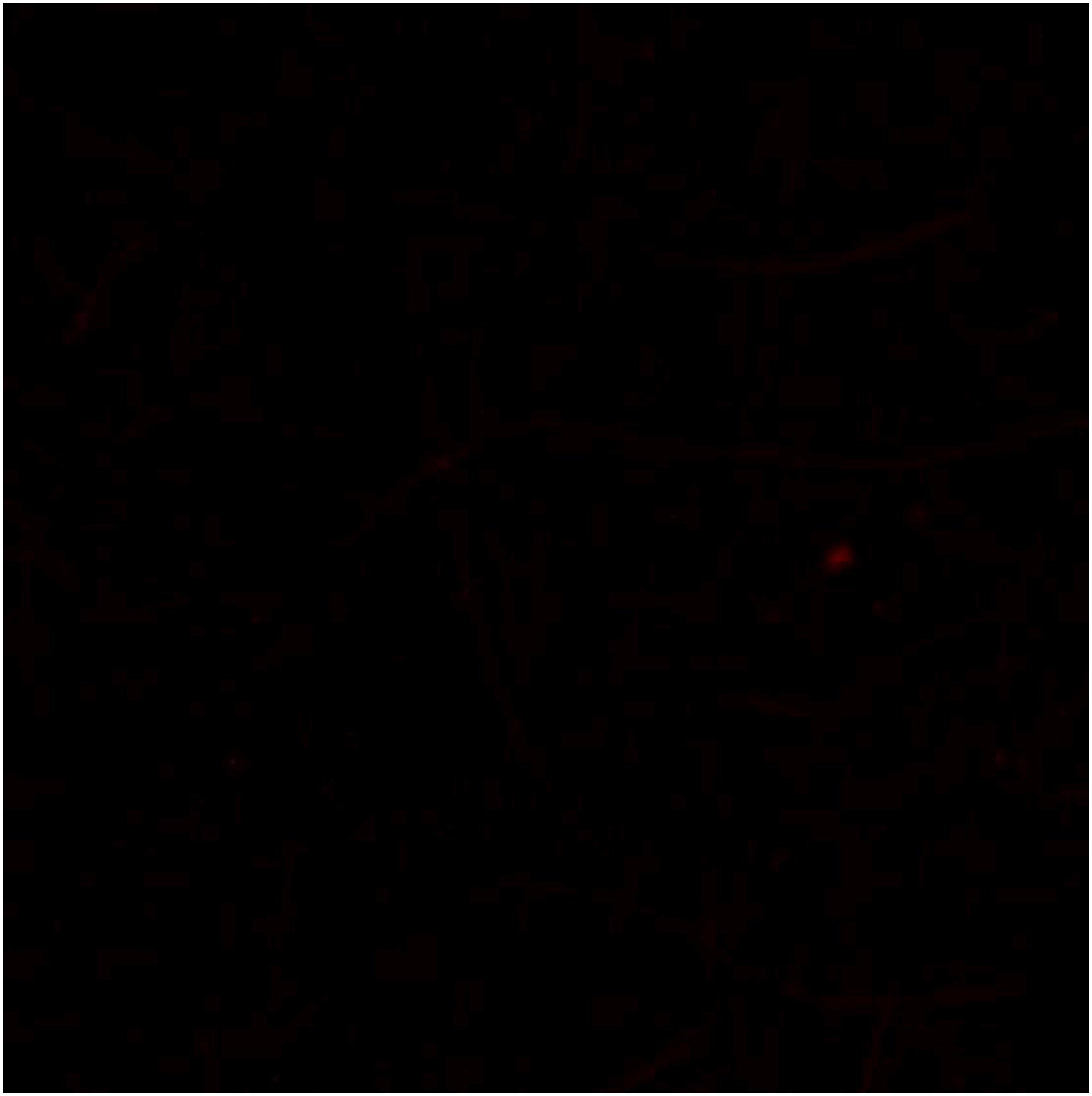

Fig. S14. Confocal laser scanning microscopy of CTMP sheet. (λ = 561 nm).

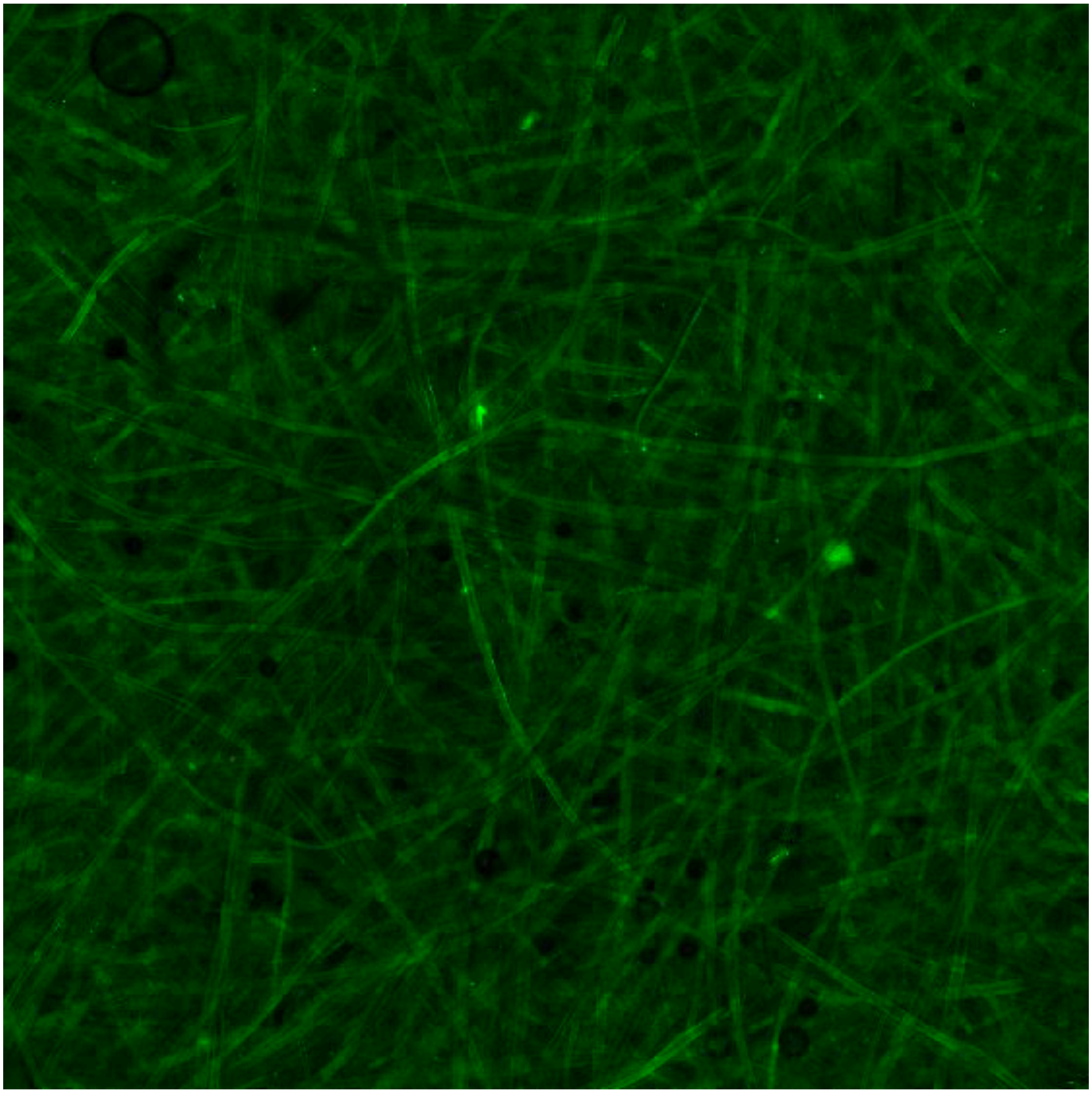

Fig. S15. Confocal laser scanning microscopy of CTMP sheet. (λ = 405 nm).

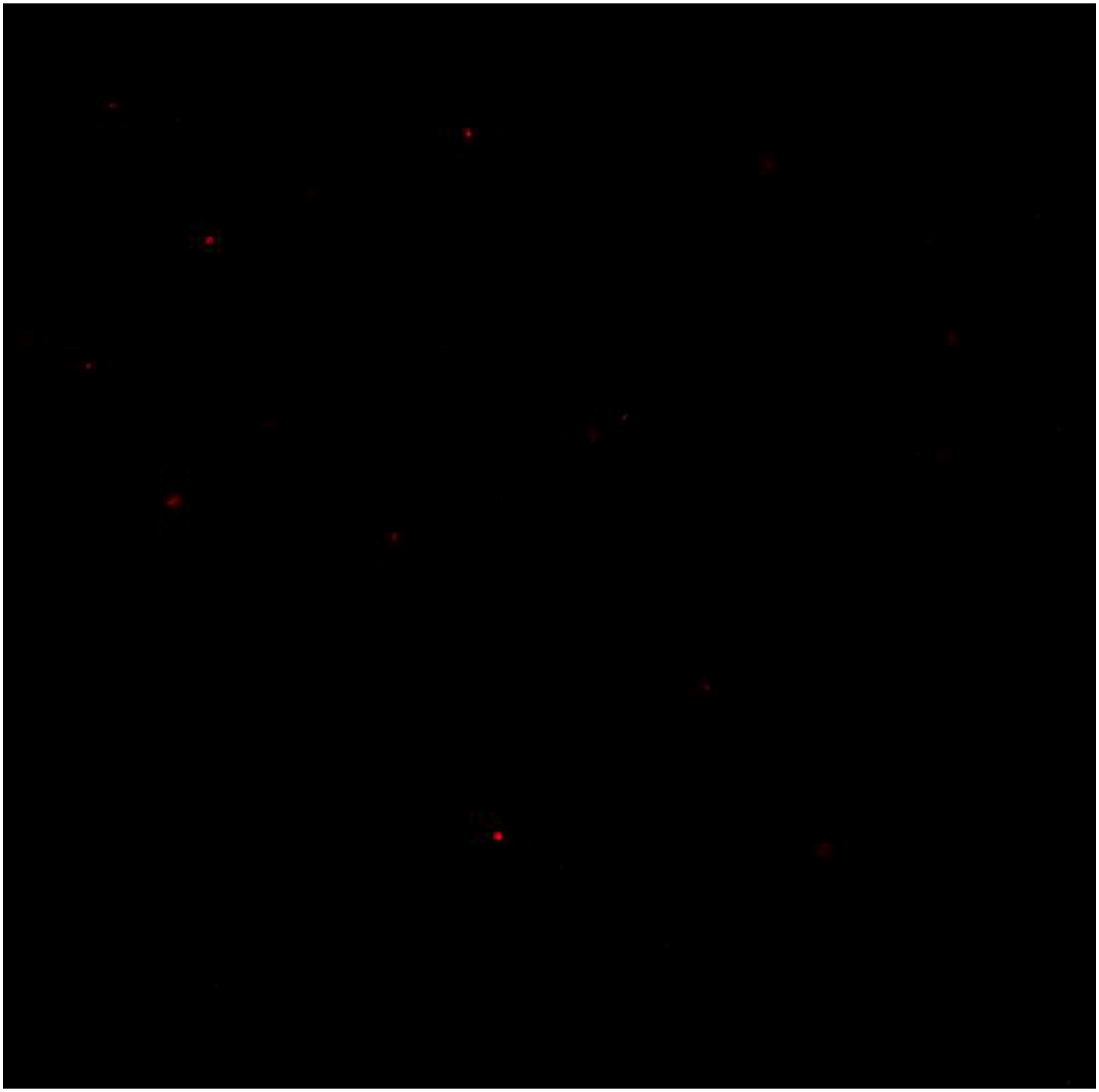

Fig. S16. Confocal laser scanning microscopy of CA-treated (0.6 wt%) CS-TGA-TAMRA,CMC-BSP sheet. (λ = 561 nm).

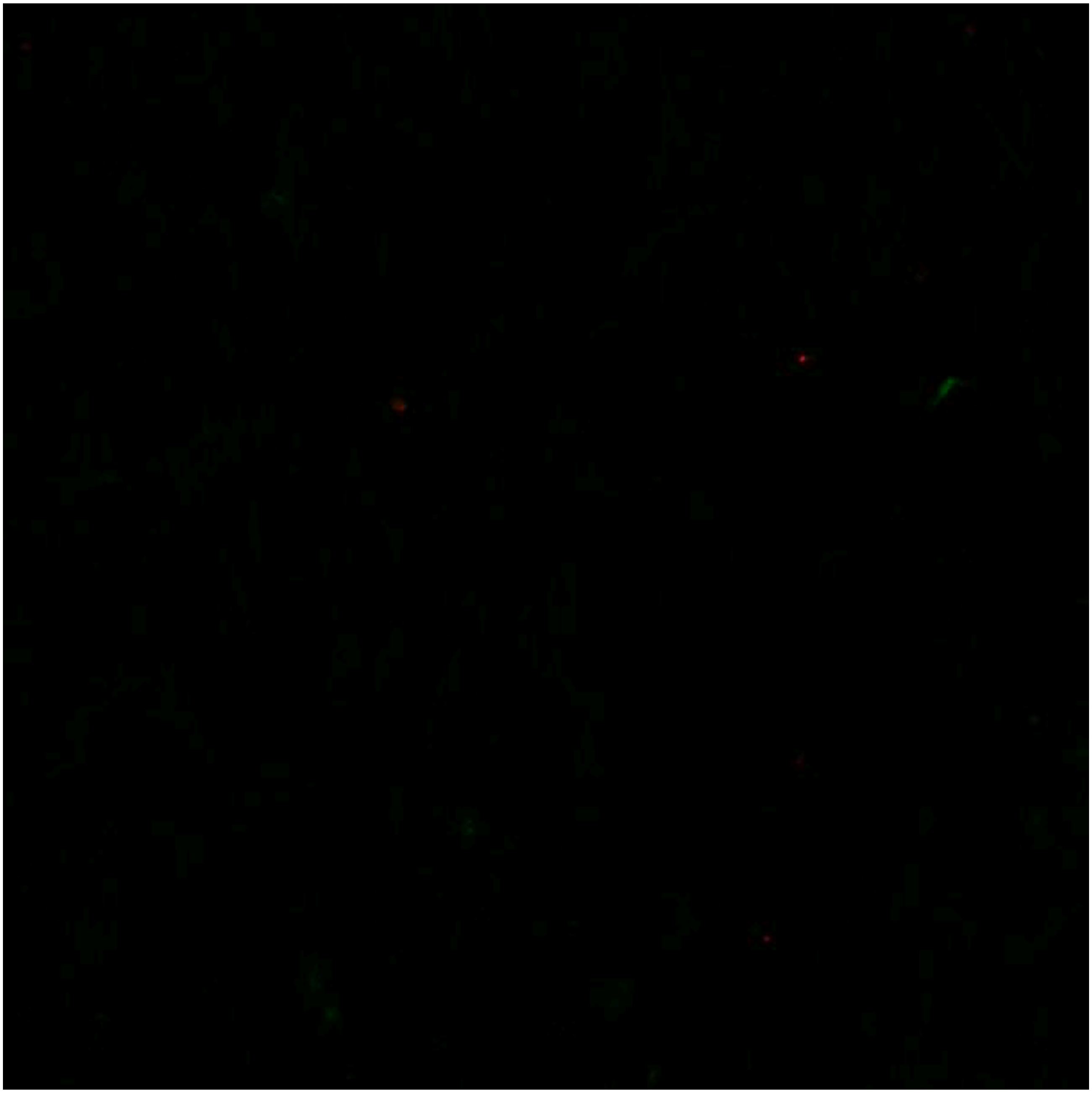

Fig. S17. Confocal laser scanning microscopy of CS-TGA-TAMRA,CMC-BSP sheet. (λ = 561 nm).

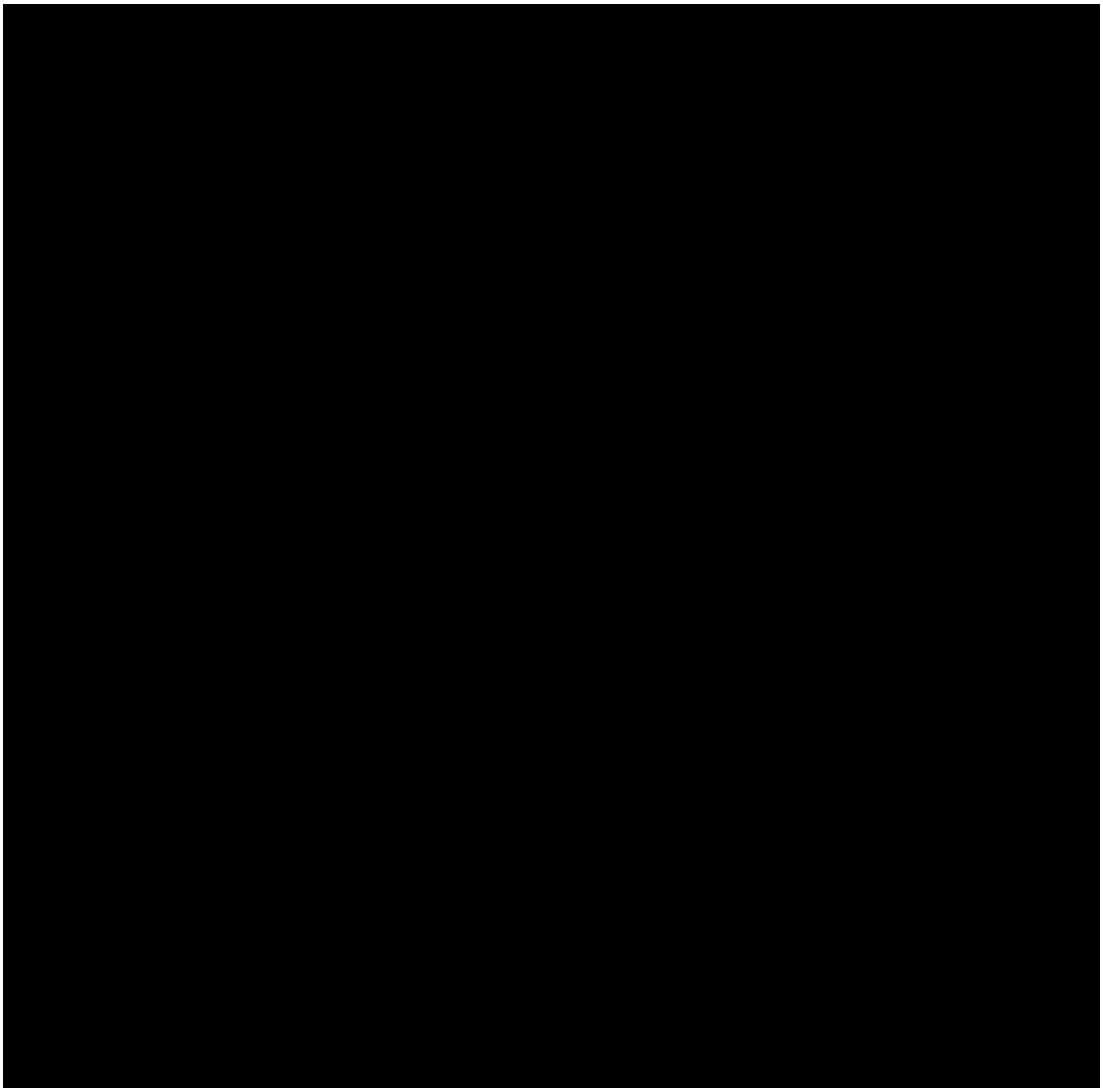

Fig. S18. Confocal laser scanning microscopy of BSP sheet. (λ = 561 nm).

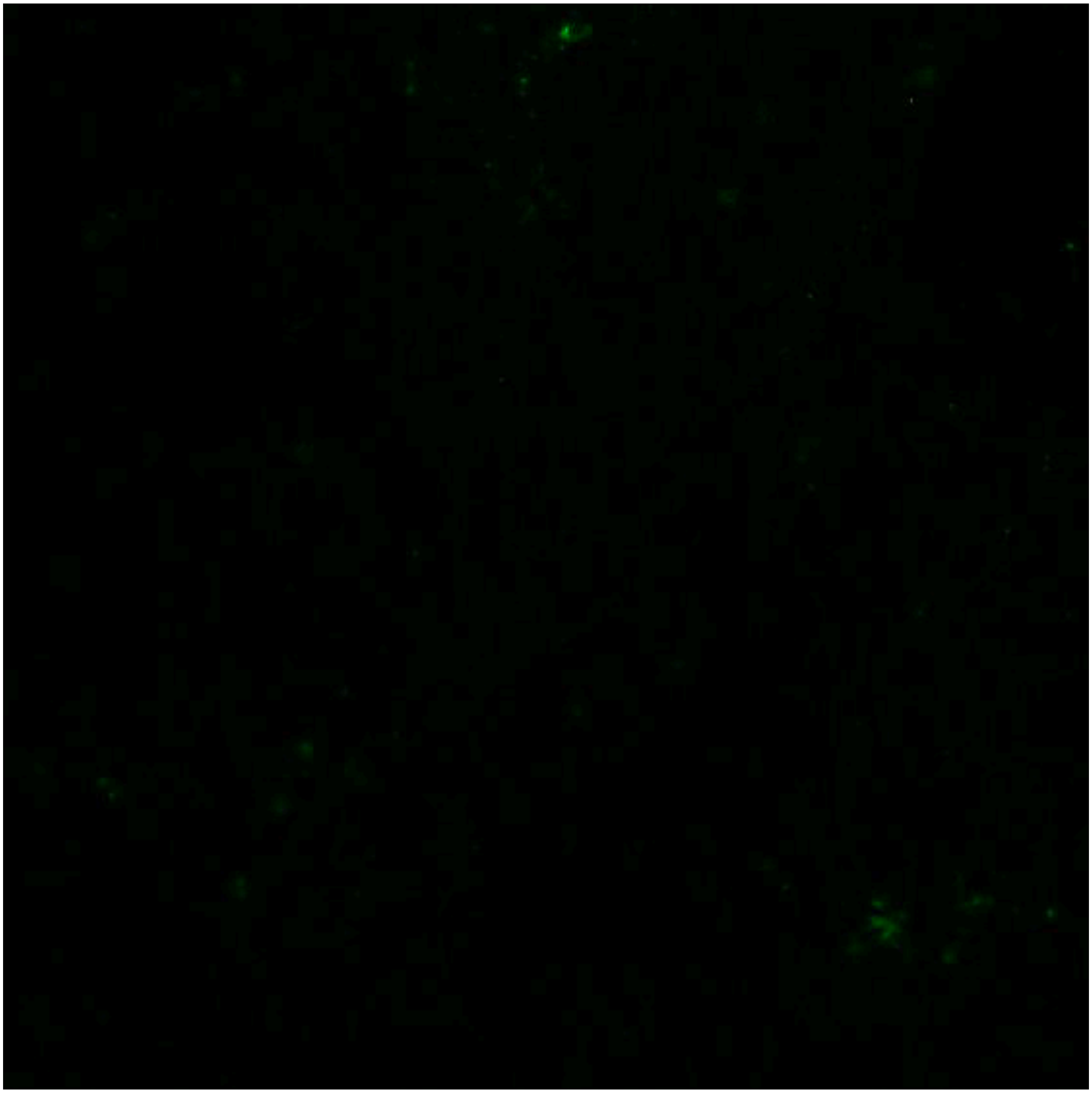

Fig. S19. Confocal laser scanning microscopy of BSP sheet. (λ = 405 nm).

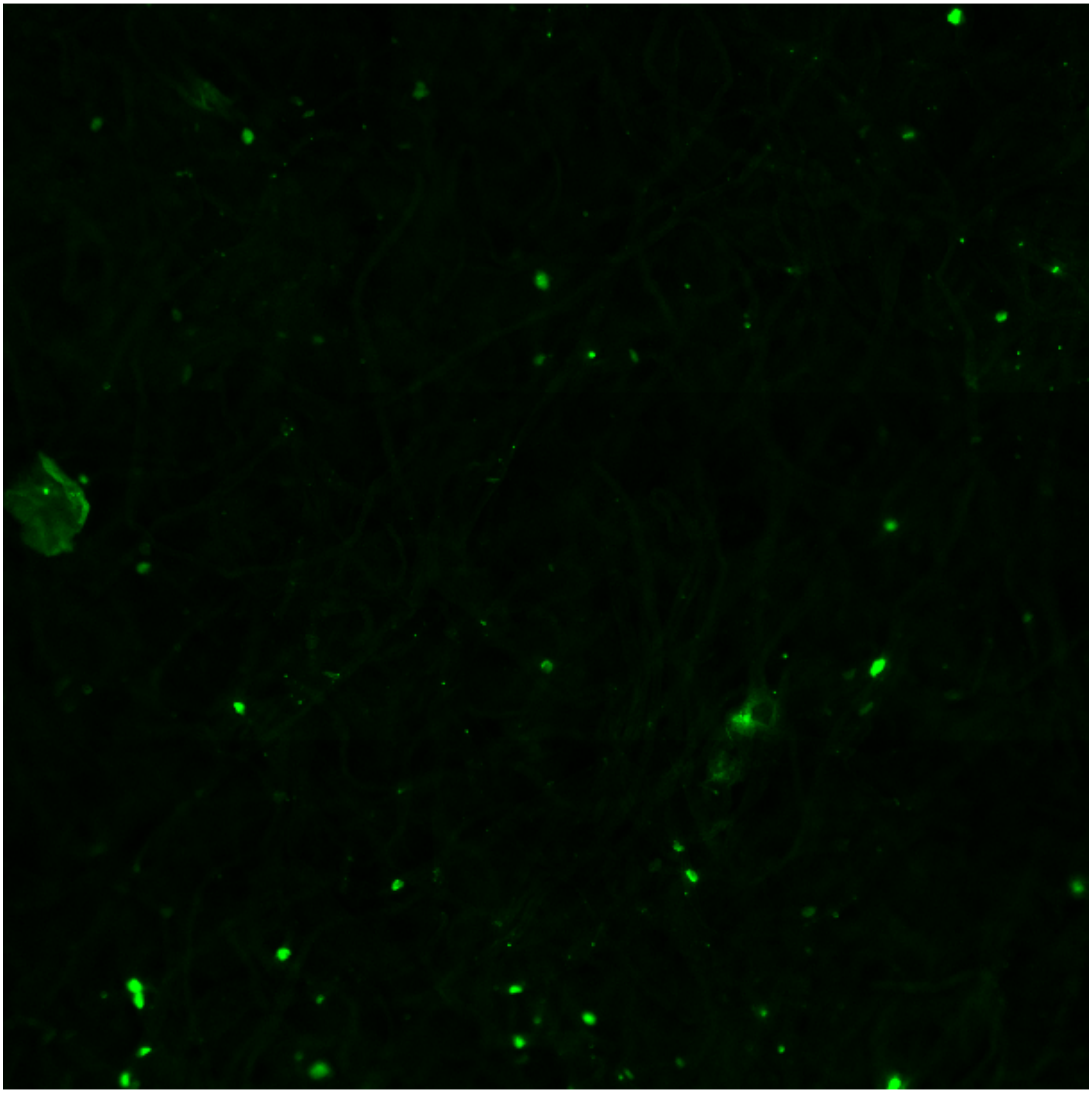

Fig. S20. Confocal laser scanning microscopy of CA-treated (0.6 wt%) CS-TGA-quinidine,CMC-BSP sheet. (λ = 405 nm).
